# Nitrogen Mineralization of a Loam Soil Supplemented with Organic–Inorganic Amendments under Laboratory Incubation

**DOI:** 10.3389/fpls.2016.01038

**Published:** 2016-07-21

**Authors:** M. Kaleem Abbasi, Abdul Khaliq

**Affiliations:** Department of Soil and Environmental Sciences, Faculty of Agriculture, The University of Poonch Rawalakot, RawalakotPakistan

**Keywords:** mineralization, nitrification, N transformations, organic amendments, poultry manure, wheat straw residues

## Abstract

The quantification of nitrogen (N) supplying capacity of organic amendments applied to a soil is of immense importance to examine synchronization, N release capacity, and fertilizer values of these added materials. The aims of the present study was to determine the potential N mineralization and subsequent nitrification of separate and combined use of poultry manure (PM), wheat straw residues (WSR), and urea N (UN) applied to a loam soil incubated periodically over 140 days period. In addition, changes in total soil N and carbon contents were also monitored during the study. Treatments included: PM_100_, WSR_100_, PM_50_ + WSR_50_, UN_100_, UN_50_ + PM_50_, UN_50_ + WSR_50_, UN_50_ + PM_25_ + WSR_25_, and a control (unfertilized). All the amendments were applied on an N-equivalent basis at the rate of 200 mg N kg^-1^. Results indicated that a substantial quantity of N had been released from the added amendments into the soil mineral pool and the net cumulative N mineralized varied between 39 and 147 mg N kg^-1^, lowest in the WSR and highest in the UN_50_ + PM_50_. Significant differences were observed among the amendments and the net mineral N derived from a separate and combined use of PM was greater than the other treatments. The net cumulative N nitrified (NCNN) varied between 16 and 126 mg kg^-1^, highest in UN_50_ + PM_50_ treatment. On average, percentage conversion of added N into available N by different amendments varied between 21 and 80%, while conversion of applied N into NO_3_^-^–N ranged between 9 and 65%, and the treatment UN_50_ + PM_50_ displayed the highest N recovery. Urea N when applied alone showed disappearance of 37% N (N unaccounted for) at the end while application of PM and WSR with UN reduced N disappearance and increased N retention in the mineral pool for a longer period. Organic amendments alone or in combination with UN improved organic matter buildup and increased soil N concentration. These results demonstrate the existence of substantial amounts of N reserves present in PM and WSR that can be utilized efficiently and effectively as potential N source for the management of nutrient poor soils and plant growth.

## Introduction

The mountainous and hilly region of the Kashmir State is characterized by undulating topography, steep slopes, and high annual rainfall. As a result the productivity of cultivated land is severely affected by accelerated erosion and land degradation (Naz and Romshoo, 2012). The authors reported that the extent of soil erosion in the valley of Kashmir (Indian part) indicated that 48.3% of the area is under very high erosion risk. Soil erosion in the mountain ecosystems has been unprecedented in speed and scale over the past decades resulting in drastic ecological changes in the region including organic matter loss, soil fertility depletion, water erosion, and environmental degradation (Tiwari et al., 2008). Under such situation, agriculture production can be achieved by applying high nitrogen (N) fertilization. However, the hilly and sloping landscape with high rainfall may cause inefficient utilization of an expensive input because of surface runoff and leaching losses (Tiwari et al., 2010). Use of organic materials as soil amendments may be an important management strategy for restoration of these degraded soils in addition to overall improvement in the physical, chemical, and microbiological environments of the soil (Zingore et al., 2008; Abbasi and Khizar, 2012; Abbasi and Tahir, 2012; Khaliq and Abbasi, 2015a).

Among manures, poultry manure (PM) is easily available because the poultry industry is one of the largest and fastest growing agro-based industries in the region (Bolan et al., 2010). The application of PM/litter to crop lands is important as an environmentally friendly means of waste disposal and also as a valuable source of plant nutrients to maintain or restore soil fertility (Bolan et al., 2010). Application of PM to soil can supply all important nutrients to agricultural crops including N, P, K, Ca, and Mg in amounts sufficient to support normal growth (Tewolde et al., 2005, 2010). Previous work has shown that the chemical composition of animal manures (N and C) can affect the amount of net N mineralized from manures (Sistani et al., 2008). An incubation study in soil amended with PM, cattle manure (CM), and urea fertilizer (UF) indicated that PM amended soils had significantly higher total N mineralization capacity (244 kg ha^-1^) than CM (109 kg ha^-1^), UF (138 kg ha^-1^), and CM + UF (141 kg ha^-1^) treated soils (Alizadeh et al., 2012).

Similar to PM, wheat (*Triticum aestivum*) straw residue (WSR) is an agricultural by-product that plays an important role in organic matter buildup and fertility status of poor and degraded soils (Khan and Mubeen, 2012). This can be accomplished by adding it alone or by mixing with mineral N fertilizer. For this purpose, one of the crucial factors is an accurate estimation of the N immobilization and re-mineralization rates following WSR application (Corbeels et al., 2000). Previous studies indicated that WSR induced net N immobilization during initial stages, and released N at later stages largely depending on several biotic and abiotic factors (Corbeels et al., 2000; Roy et al., 2011). It has been reported that application of crop residues reduces N losses due to slower cycling pool that caused greater N retention in soil amended with crop residue (73%) than with fertilizer N (26%; Delgado et al., 2010).

The present study was conducted with the hypothesis that application of PM and WSR will be able to (i) release substantial N into the soil mineral N pool for both short and long term period and as such may be considered as a value-added N nutrient source, and (ii) both organic sources when combined with urea N may decrease the rate of mineralization of UN, and increase the retention of available N in soil for a longer period of time. The objectives of the present study were to quantify the relative rates of N mineralization and subsequent nitrification of PM, WSR, and urea N applied alone or in different combinations to a soil incubated for different time intervals and also examine the relative changes in soil total N and C in response to added amendments under controlled environmental conditions.

## Materials and Methods

### Sites Description

The study site (Rawalakot) is located at latitude 33°51′32.18′′N, longitude 73° 45′34.93′′E and an elevation of 1638 m above the sea level, in the north–east of Pakistan under the foothills of great Himalayas district Poonch Azad Jammu and Kashmir, Pakistan. The soil in the study sites was loam in texture, classified as a Humic Lithic Eutrudept (Ali et al., 2006). The climate of the region is sub-temperate. Mean daily maximum and minimum air temperatures ranged from 27 to 29°C (June–July) and 1.0 to -3.5°C (January–February). The mean (average of 3 years, 2009–2011) annual rainfall was 1,388 mm with 45% of the total precipitation during June–September and 43% during January–April. Details of the study site and the geographical map of the area are described in Khaliq and Abbasi (2015a,b).

### Soil Sampling/Collection

Surface bulk soil (0 to 15 cm) from a field under long-term wheat–maize management system was collected from the Faculty of Agriculture, The University of Poonch, Rawalakot Azad Jammu and Kashmir, Pakistan. Soil was collected from five different points of the field by soil auger and mixed as one composite sample. Before sampling, grass, forest litter or any other material on the soil surface were removed. After being brought into the laboratory, the field-moist soil was passed through a 4–mm sieve to eliminate coarse rock and plant material, thoroughly mixed to ensure uniformity and stored at 4°C prior to use. A subsample of about 500 g was taken, air dried (in shade), and passed through a 2–mm sieve and used for the determination of selected soil physical and chemical characteristics (**Table 1**).

**Table 1:** The initial physical and chemical characteristics of soil used in the incubation study.

Soil properties	Values
Bulk density (g cm^-3^)	1.32 (±0.03)
Sand (g kg^-1^)	433.9 (±20.3)
Silt (g kg^-1^)	326.0 (±18.8)
Clay (g kg^-1^)	240.1 (±11.5)
Textural class	Loam
Soil pH (1:2.5H_2_O)	6.89 (±0.17)
Organic matter (g kg^-1^)	10.3 (±0.78)
Organic carbon (g kg^-1^)	5.97 (±0.24)
Total N (g kg^-1^)	0.53 (±0.02)
NH^+^_4_-N (mg kg^-1^)	8.85 (±0.52)
NO^-^_3_-N (mg kg^-1^)	7.21 (±0.26)
Available P (mg kg^-1^)	5.49 (±0.31)
Available K (mg kg^-1^)	98.5 (±7.02)
Iron (Fe; mg kg^-1^)	17.8 (±1.02)
Manganese (Mn; mg kg^-1^)	6.2 (±0.43)
Zinc (Zn; mg kg^-1^)	8.4 (±0.47)
Copper (Cu; mg kg^-1^)	3.79 (±0.32)
Cation exchange capacity (CEC) cmol^(+)^ kg^-1^soil	11.9 (±1.72)

*values in the parenthesis are the standard error of means (SEM), i.e., *n* 3.*

### Collection of Poultry Manure and Wheat Straw Residues

Poultry manure was collected directly from a local poultry farm located at Trar, Rawalakot, while wheat straw was collected from the wheat flour mill located nearby Rawalakot at Goinalla, Azad Jammu and Kashmir. A composite sample of well-rotted PM was air dried, crushed into smaller particles by hand pressing, homogenized, and passed through a 1–mm sieve. Similarly, wheat straw residues (WSR) were dried in an oven at 70°C for overnight, milled, and sieved through a 1–mm sieve. Samples of both organic materials were stored (4°C) in tightly closed plastic containers before use. Triplicate samples from each of PM and WSR were analyzed for total N, total carbon (C), P, and K. For organic C and K estimation, 5 g of dried PM and WSR samples were ignited in a muﬄe furnace at 500°C for three to 5 h (Ball, 1964; Okalebo et al., 1993). Burnt (ashed) materials were extracted by repeatedly (three times) using 50% hydrochloric acid (HCl), and K was measured from the extractant (dissolved material) by flame photometric. For N and P colorimetric determinations, the samples were digested (wet digestion) by using sulfuric acid (H_2_SO_4_) and hydrogen peroxide (H_2_O_2_) (Okalebo et al., 1993). Total N of the WSR was determined by digesting a 0.5 g dried residue sample in the presence of both H_2_SO_4_ and H_2_O_2_ (5 and 7–mL). The contents were heated in a digestion tube at 400°C for 1 h. The lignin content was determined using Van Soest methods (Van Soest et al., 1991). Soluble polyphenols were extracted in hot water (100°C for 1 h) and determined by colorimetery using Folin-Denis reagent (Folin and Denis, 1915). The chemical composition of both PM and WSR has been described in **Table 2**.

**Table 2:** Chemical composition of organic amendments, i.e., poultry manure and wheat straw residues (WSRs) used in the study.

Chemical properties	Poultry manure (PM)	Wheat straw residues (WSR)
Total nitrogen (g kg^-1^)	25.7 ± 3.1	9.1 ± 1.4
Total phosphorus (g kg^-1^)	16.1 ± 1.4	0.58 ± 0.1
Total potassium (g kg^-1^)	18.1 ± 1.6	11.4 ± 0.6
Total carbon (g kg^-1^)	349.2 ± 23.5	418.0 ± 23.1
C:N	13.2 ± 1.7	46.0 ± 2.3
Organic matter (g kg^-1^)	602.5 ± 24.8	586.2 ± 21.5
Calcium (g kg^-1^)	35.0 ± 4.0	2.9 ± 0.3
Magnesium (g kg^-1^)	6.2 ± 0.82	1.7 ± 0.1
Iron (mg kg^-1^)	1293.0 ± 106.8	97.6 ± 12.7
Zinc (mg kg^-1^)	428.2 ± 24.4	17.4 ± 3.0
Manganese (mg kg^-1^)	641.5 ± 19.6	65.8 ± 8.3
Copper (mg kg^-1^)	578.0 ± 20.7	14.5 ± 2.5
Cellulose (g kg^-1^)	67.9 ± 2.8	429.0 ± 15.1
Hemicellulose (g kg^-1^)	— — — —	198.4 ± 10.7
Lignin (g kg^-1^)	58.4 ± 3.3	127.0 ± 11.5
Polyphenol (mg kg^-1^)	208.7 ± 2.5	43.1 ± 2.3

*Values presented after ± are standard error of the means (SEM), *n* = 3.*

### Laboratory Incubation

A known weight of fresh soil samples (100 g, oven dry weight basis) already stored in the refrigerator (not more than 15 days’ time) was transferred into 500–mL glass jars. Moisture content of soil was adjusted to 60% of its water holding capacity by adding deionized water and pre-incubated at 25°C for 1 week prior to actual incubation to stabilize the microbial activity. The experiment comprised of two main factors, i.e., treatments and time intervals. Treatments included: unfertilized control, poultry manure full (PM_100_), wheat straw residues full (WSR_100_), urea N full (UN_100_), PM half and WSR half (PM_50_ + WSR_50_), UN_50_ + PM_50_, UN_50_ + WSR_50_, and UN_50_ + PM_25_ + WSR_25_ (**Table 3**). Time intervals involved 14 incubation periods, i.e., 0, 1, 2, 4, 6, 7, 14, 21, 28, 35, 49, 63, 84, 105, and 140 days (after amendments application). The treatment combinations were applied three times. Altogether, a total of 316 experimental units were used at the start of the experiment. The subscripted values used (i.e., 100, 50, and 25) on each source (PM, WSR, and UN) refer to the percentage of total N applied from each source. All the amendments were applied on an N-equivalent basis at the rate of 200 mg N kg^-1^. The summary of different treatments has been described in **Table 3**. Phosphorus and K were also added to each jar (including control) at the rate of 90 mg P kg^-1^ and 60 mg K kg^-1^ as single super phosphate and sulfate of potash, respectively. Following the addition of all amendments, soil was thoroughly mixed, and the weight of each jar was recorded. Jars were covered with parafilm that was perforated with a needle to ensure natural gas exchange. All the amended jars were kept in an incubator at 25 ± 2°C for a total of 140 days. Jars were arranged in the incubator according to completely randomized design. Soil moisture was checked/adjusted every 2 days by weighing the glass jars and adding the required amount of distilled water when the loss was greater than 0.05 g. During this process, care was taken not to disturb the soil, either through stirring or shaking.

**Table 3:** Summary of the different experimental treatments/units used in the experiment.

Treatments	N from urea N (UN)	N from poultry manure (PM)	N from wheat straw residues (WSR)
Control	0	0	0
PM_100_	0	200 mg N kg^-1^	0
WSR_100_	0	0	200 mg N kg^-1^
PM_50_ + WSR_50_	0	100 mg N kg^-1^	100 mg N kg^-1^
UN_100_	200 mg N kg^-1^	0	0
UN_50_ + PM_50_	100 mg N kg^-1^	100 mg N kg^-1^	0
UN_50_ + WSR_50_	100 mg N kg^-1^	0	100 mg N kg^-1^
UN_50_ + PM_25_ + WSR_25_	100 mg N kg^-1^	50 mg N kg^-1^	50 mg N kg^-1^

*The subscripted values used (i.e., 100, 50, and 25) on each source (PM, WSR, and UN) refer to the percentage of total N applied from each source.*

### Soil Extraction and Analysis

Samples of all treatments incubated for different intervals were analyzed for total mineral nitrogen (TMN) and ammonium-N (NH_4_^+^–N). Initial concentration of TMN and NH_4_^+^–N at day zero was determined by extracting soil samples with 200 ml of 2 M KCl added directly to the flask immediately after incorporation of each amendment. Thereafter, triplicate samples from each treatment were removed randomly from the incubator at different incubation periods and extracted by shaking for 1 h with 200 ml of 2 M KCl followed by filtration through Whatman’s No. 40 filter paper. The mineral N-contents of the extract were determined by using the steam distillation and titration method (Keeney and Nelson, 1982). Nitrate–N was calculated by subtracting NH_4_^+^–N from total mineral N the procedure/method used in our previous studies (Abbasi and Adams, 2000; Abbasi et al., 2001; Abbasi and Khizar, 2012). Any nitrogen dioxide (NO_2_) present would have been included in NO_3_^-^ fraction. However, the presence of any known bias with high N_2_O-N values that could inflate their NO_3_-N calculations.

The C and N contents (total C and total N) of soil was also monitored during incubation. For this purpose, a 10 g subsample of incubated soil was removed from each jar at each sampling period and placed in tared petri dishes to determine changes in total N and C with time. Soil organic carbon (SOC) was determined according to Nelson and Sommers (1982) while soil total N (STN) was determined by the Kjeldahl method (Bremmer and Mulvaney, 1982). Net cumulative N mineralized (NCNM) expressed as N mineralization (or total mineral N; TMN) and net cumulative N nitrified (NCNN) articulated as NO_3_^-^–N accumulation at each sampling time (*t*) was calculated as described earlier (Griffin et al., 2005).

### Statistical Analysis

The data were subjected to analysis of variance technique (ANOVA) using Statistix v. 8.1 software package by Repeated Measures Design. Tukey’s honest significant difference (HSD) test was used for multiple comparisons among treatments at the same time interval and among time intervals within the same treatment at *p* ≤ 0.05. Treatments were taken as the between-subject factor and time as the within-subject factor. Correlations between total mineral N (N mineralization) and SOC, and between TMN and STN were calculated using SPSS 12 for Windows^1^.

## Results and Discussion

### Nitrogen Mineralization of Added Amendments

There were significant (*p* ≤ 0.05) differences among N treatments (N), time periods (T), and their interactions (N × T) for NCNM and NCNN, and changes in soil organic N (SON; **Table 4**). Treatment and time were also significant for changes in soil organic C (SOC).

**Table 4:** Analysis of Variance (ANOVA) for changes in net cumulative nitrogen mineralized (NCNM), net cumulative N nitrified (NCNN), soil organic carbon (SOC), and soil total N (STN) of the soil amended with PM, WSR, urea nitrogen (UN), and their combinations (at the rate equivalent to 200 mg N kg^-1^ soil) over different incubation timings till 140 days period.

Variables	NCNM	NCNN	SOC	STN
N Treatments (N)	**	**	**	**
Time periods (T)	**	**	**	**
N × T	**	**	NS	**

**Represents highly significant (*P* ≤ 0.01); NS, non-significant.

**FIGURE 1:**
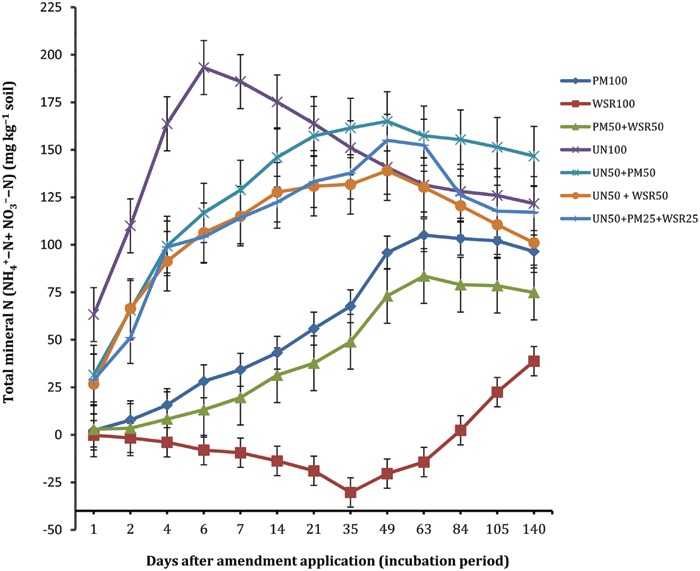
**Changes in net cumulative N mineralized (NCNM) in response to the application of poultry manure (PM), wheat straw residues (WSR), and urea N (UN) applied alone or in different combinations (at the rate equivalent to 200 mg N kg^-1^ soil) to a soil incubated at 25°C for 140 days under controlled laboratory conditions.** Vertical lines on each bar represent the Tukey’s HSD (*p* ≤ 0.05) showing significance level of each treatment at different incubation periods. The subscripted values used (i.e., 100, 50, and 25) on each source (PM, WSR, and UN) refer to the percentage of total N applied from each source.

The N mineralization of the soil amended with PM (PM_100_) was almost negligible at the start (2.3 mg kg^-1^), but progressively increased with time, and the highest concentration of 105.1 mg kg^-1^ was recorded at day 63 (**Figure 1**). Thereafter, the concentration remained statistically unchanged. At the end (day 140), 96.5 mg N kg^-1^ was recorded indicating that a total of 48% of N applied from PM was released into the mineral N pool. This is within the range found in earlier studies, e.g., 51 and 53% (Qafoku et al., 2001), 25 to 61% (Grijalva et al., 2010), 61% (Alizadeh et al., 2012), 44% (Moore et al., 2010) and 42% (Shah et al., 2013). The variability in the reported values may be due to the variability in chemical composition of PM, the feed/diet effect, the proportion of litter to droppings, the manure handling system, and the type of litter (Bolan et al., 2010).

The N mineralization from WSR displayed negative values (-0.2 to -30.3 mg kg^-1^) between days 1 and 63, reflecting net immobilization. The highest immobilization (-30.3 mg kg^-1^; 15.2% of added N) was observed at day 35. However, from days 84 to 140, positive values of 2.4 to 38.8 mg kg^-1^ were observed showing net mineralization of 19.4% of the total N applied. The complex biochemical composition of crop residues with high C:N are responsible for net immobilization initially found. It has been reported that plant/crop residues of C:N wider than 20:1 are immobilized faster than being mineralized (Mubarak et al., 2001). The C:N of WSR used in this study was 46:1; therefore, immobilization was most likely to occur during decomposition and transformation processes as reported previously in soil supplemented with wheat crop residues of high C:N (75:1; Mubarak et al., 2001). Generally, incorporation of straw residues induces net N immobilization during initial decomposition stages and releases N at a later stage within a few weeks’ time (Corbeels et al., 2000; DeNeve et al., 2004; Reddy et al., 2008).

The soil amended with the combined treatment PM_50_ + WSR_50_ released a total of 37% N applied, lower than that recorded from PM (48%), but substantially higher than that of WSR (19%). In contrast to WSR_100_ treatment, PM_50_ + WSR_50_ displayed net positive values of TMN throughout the incubation, indicating that immobilization from WSR (as observed in WSR_100_) was offset by the N mineralized from PM. Previous studies indicated higher N mineralization where both organic amendments (*Sesbania* and WSR) were combined, compared to their sole applications, indicating synergistic relationships between the two inputs (Palm et al., 2001; Singh et al., 2007).

The soil amended with urea N (UN) exhibited the highest N mineralization during the initial phase, and the maximum N release of 193.3 mg kg^-1^ was observed at day 6. Thereafter, the concentration tended to decline with time, most likely because of simultaneous nitrification-denitrification processes in the system (Abbasi and Adams, 2000) in addition to possible chances of immobilization (Abbasi et al., 2001). Expressed as percentage of total N applied, 85% of N was hydrolyzed during the first 6 days of incubation. The NCNM from the combined treatments UN_50_ + PM_50_, UN_50_ + WSR_50_, and UN_50_ + PM_25_ + WSR_25_ were significantly higher than the sole application of PM and WSR, and at par with or higher than that recorded from UN_100_ (during later part of the incubation). At the end of incubation (day 140), the NCNM released from UN_50_ + PM_50_ was significantly higher than the NCNM released from the remaining treatments, including UN_100_. These results indicated that addition of N from mineral fertilizers accelerates the decomposition of organic materials and has positive effects on belowground N transformation processes.

### Accumulation of Nitrate-Nitrogen

**FIGURE 2:**
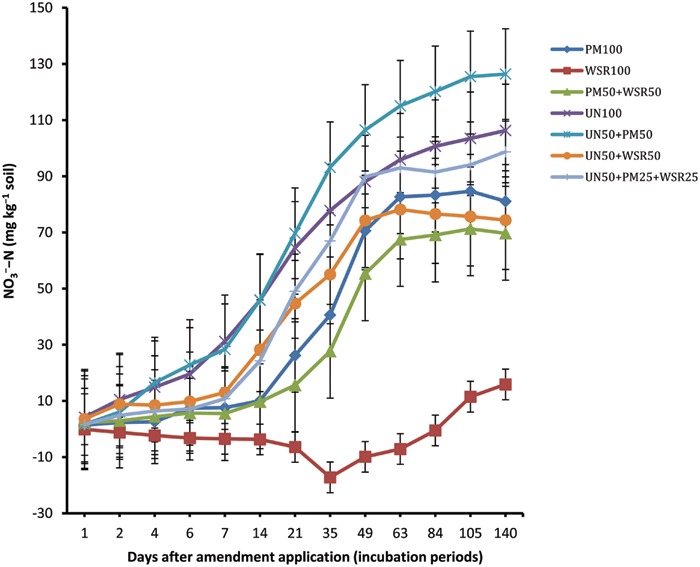
**Changes in net cumulative N nitrified (NCNN) in response to the application of poultry manure (PM), wheat straw residues (WSR), and urea N (UN) applied alone or in different combinations (at the rate equivalent to 200 mg N kg^-1^ soil) to a soil incubated at 25°C for 140 days under controlled laboratory conditions.** Vertical lines on each bar represent the Tukey’s HSD (*p* ≤ 0.05) showing significance level of each treatment at different incubation periods. The subscripted values used (i.e., 100, 50, and 25) on each source (PM, WSR, and UN) refer to the percentage of total N applied from each source.

The NCNN displayed similar trend to that recorded for NCNM (**Figure 2**). Among different amendments, soil amended with UN (UN_100_) and UN_50_ + PM_50_ exhibited the highest NO_3_^-^–N accumulation, significantly higher than (*p* ≤ 0.05) the NO_3_^-^–N recorded from the remaining treatments (for most of the incubation periods). The difference between UN_100_ and UN_5_0 + PM_50_ was non-significant in general. Among organic amendments, WSR_100_ showed negative values until day 84, and thereafter concentration increased to a maximum of 15.9 mg kg^-1^ at day 140 (i.e., 8% of total N applied). On the other hand, soil amended with PM_100_ and PM_50_ + WSR_50_ exhibited a substantial nitrification potential of 81.1 and 69.7 mg kg^-1^, respectively, at the end of incubation, indicating 41 and 35% of total N applied being nitrified (i.e., 84 and 93% of the total NCNM).

The accumulation of NO_3_^-^–N in UN amended soil significantly increased during incubation and the highest concentration of 106.3 mg kg^-1^ was recorded at the end (day 140), showing that 53% of applied N from UN was nitrified. The gain in NO_3_^-^–N with respect to total NCNM was 87%. The pattern of NO_3_^-^–N accumulation in soil amended with combined treatments (UN_50_ + PM_50_, UN_50_ + WSR_50_, and UN_50_ + PM_25_ + WSR_25_) exhibited a significant increase in NO_3_^-^–N compared to the sole application of these amendments. At the end, UN_50_ + PM_50_ exhibited the highest NO_3_^-^–N accumulation of 126.4 mg kg^-1^ compared to other treatments including UN_100_ (i.e., 106.3 mg kg^-1^). The accumulation of NO_3_^-^–N by the combined treatments UN_50_ + PM_50_, UN_50_ + WSR_50_, and UN_50_ + PM_25_ + WSR_25_ in relation to total N applied was 63, 37, and 49%, respectively, while the accumulation in relation to total NCNM was 86, 74, and 84%. Averaged across incubation timings, accumulation of NO_3_^-^–N in different treatments was in the order UN_50_ + PM_50_ > UN_100_ > UN_50_ + PM_25_ + WSR_25_ > UN_50_ + WSR_50_ = PM_100_ > PM_50_ + WSR_50_ > WSR_100_.

Our results indicated that accumulation of NO_3_^-^–N in the mineral N pool was significantly increased when PM was combined with WSR (PM_50_ + WSR_50_) or when UN was combined with PM and WSR (UN_50_ + PM_25_ + WSR_25_), compared to the sole application of WSR (WSR_100_). The low nitrification of added WSR was due to its low mineralization capacity, as both mineralization and nitrification in the preset study represented a significant correlation between each other (*r* = 0.9). In our previous study, accumulation of NO_3_^-^–N under PM, white clover (*Trifolium repens*) residues (WCR), and PM + WCR was 16, 20, and 38%, respectively, that had been significantly increased to 55, 63, and 69% when UN was combined with these amendments (Abbasi and Khizar, 2012). The authors explained that the increasing nitrification in the integrated treatments was due to the increasing microbial biomass C and N that may have affected the microbial activity, thereby improving N transformation processes, including nitrification. Under field conditions, application of mineral N fertilizers in any form subjected to losses via denitrification, runoff, leaching etc., in addition to that taken up by the plants. The mineral N fertilizer losses can be minimized by applying mineral N with organic N sources. Application of mineral N with organic sources slows down net N mineralization as soil microbes immobilize mineral N to decompose the organic sources. As a result the NH_4_^+^-N remained stable for longer period in the mineral pool and chances of nitrification will be delayed and consequently N losses, especially denitrification and leaching, will be minimized.

### Changes in Soil Total Nitrogen

All N sources significantly (*p* ≤ 0.05) increased soil total N (STN) compared to the control (**Table 5**). The pattern of changes in STN over different incubation periods was similar to that recorded for SOC (i.e., decreasing over time). The STN unaccounted for during incubation may be due to continuous mineralization of added materials or subjected to various kinds of losses during N transformations processes (Abbasi and Adams, 2000). The highest total N at different incubation timings was observed in the treatments under WSR_100_ or PM_50_ + WSR_50_. Averaged across different incubation periods, the STN in the control soil was 0.51 g kg^-1^. Soil amended with different amendments all significantly increased STN that varied between 0.57 and 0.71 g kg^-1^ (**Table 6**) representing a relative increase of 12 to 39% over the control treatment. The highest N contents were observed in the soil under WSR_100_, followed by PM_50_ + WSR_50_, UN_50_ + WSR_50_, and PM_100_. The soil under UN_100_ exhibited the lowest N content of 0.57 g kg^-1^. Results indicated a significant negative correlation between TMN and STN (*r* = -0.90) demonstrating that soil N contents were associated with mineralization potential of the added materials. In other words, low mineralization (as in case of WSR) may result in high STN accumulation. Despite the application of both PM and WSR on equivalent basis (200 mg kg^-1^), differences in STN were probably due to the variations in mineralization potential between the two sources.

**Table 5:** Changes in total N of the soil supplemented with PM, WSR and urea nitrogen (UN) applied alone or their integrated use with different combinations (at the rate equivalent to 200 mg N kg^-1^ soil) incubated at 25°C for a total of 140 days periods.

Treatments	Incubation time periods (days)															
	0	1	2	4	6	7	14	21	35	49	63	84	105	140	Tukey’s HSD (*P* ≤ 0.05)
Total soil N (g kg^-1^ soil)										
Control	0.53	0.53	0.53	0.53	0.53	0.52	0.52	0.51	0.50	0.49	0.48	0.48	0.48	0.48	N.S
PM_100_	0.73	0.73	0.72	0.71	0.70	0.69	0.68	0.66	0.64	0.60	0.59	0.59	0.58	0.58	N.S
WSR_100_	0.73	0.73	0.73	0.72	0.72	0.71	0.72	0.72	0.72	0.73	0.73	0.69	0.67	0.63	N.S
PM_50_ + WSR_50_	0.72	0.72	0.72	0.71	0.70	0.70	0.68	0.67	0.66	0.63	0.62	0.61	0.61	0.61	0.11
UN_100_	0.72	0.66	0.62	0.57	0.56	0.56	0.55	0.55	0.54	0.54	0.54	0.54	0.53	0.53	0.12
UN_50_ + PM_50_	0.71	0.71	0.68	0.64	0.63	0.61	0.59	0.58	0.50	0.49	0.49	0.48	0.48	0.48	0.11
UN_50_ + WSR_50_	0.74	0.74	0.73	0.69	0.66	0.66	0.65	0.64	0.62	0.62	0.62	0.61	0.61	0.60	N.S
UN_50_ + PM_25_ + WSR_25_	0.74	0.74	0.71	0.67	0.65	0.64	0.63	0.61	0.60	0.59	0.59	0.58	0.58	0.57	0.16

Tukey’s HSD (*P* ≤ 0.05)	0.20	0.16	0.12	0.10	0.10	0.19	0.16	0.14	0.13	0.12	0.12	0.10	0.10	0.08	

*PM, poultry manure; WSR, wheat straw residues; UN, urea N. PM_*100*_, WSR_*100*_ and UN_*100*_ means application of these amendments alone with full dose at the rate of 200 mg N kg^-*1*^ soil. The subscripted values used (i.e., 100, 50, and 25) on each source (PM, WSR, and UN) refer to the percentage of total N applied from each source. The Tukey ‘s HSD (*P* ≤ 0.05) at the end of each row represent significance level among different incubation periods.*

**Table 6:** Changes in soil total nitrogen (STN) and SOC in response to the application of PM, WSR, and urea N (UN) applied alone or in different combinations to a soil incubated for 140 days under controlled laboratory conditions.

Treatments	Soil total N (g kg^-1^)	Soil organic C (g kg^-1^)
Control	0.51	6.05
PM_100_	0.66	9.09
WSR_100_	0.71	15.1
PM_50_ + WSR_50_	0.67	12.82
UN_100_	0.57	6.06
UN_50_ + PM_50_	0.58	8.14
UN_50_ + WSR_50_	0.66	10.70
UN_50_ + PM_25_ + WSR_25_	0.64	9.47
Tukey’s HSD (*P* ≤ 0.05)	0.05	1.51

*The values for each treatment are based on the average values over the incubation periods. The subscripted values used (i.e., 100, 50, and 25) on each source (PM, WSR, and UN) refer to the percentage of total N applied from each source.*

### Changes in Soil Organic Carbon

At the start of incubation (day 0), a significant (*p* ≤ 0.05) variation in SOC among different amendments was observed, and in general soil amended with organic and combined treatments displayed higher SOC compared to the control and UN_100_ treatment (**Table 7**). The highest SOC of 16.26 g kg^-1^ was recorded under WSR_100_, followed by 13.84 g kg^-1^ under PM_50_ + WSR_50_; the difference between the two was non-significant. SOC in the UN_100_ treatment was 6.52 g kg^-1^ at the start that remained consistent without any significant changes during the incubation. As PM and WSR were applied based on the N rate, the amount of organic C added to the soils from each was different. Therefore, the difference among the treatments may also because of the difference in organic C added to soils.

**Table 7:** Changes in organic carbon of the soil supplemented with PM, WSR and urea nitrogen (UN) applied alone or their integrated use with different combinations (at the rate equivalent to 200 mg N kg^-1^ soil) incubated at 25°C for a total of 140 days periods.

Treatments	Incubation time periods (days)															
	0	1	2	4	6	7	14	21	35	49	63	84	105	140	Tukey’s HSD (*P* ≤ 0.05)
Organic carbon (g kg^-1^ soil)									
Control	6.51	6.46	6.39	6.31	6.27	6.22	6.17	6.11	6.04	5.92	5.76	5.62	5.42	5.45	N.S
PM_100_	11.03	10.95	10.79	10.46	9.86	9.73	9.21	8.89	8.39	8.08	7.65	7.56	7.43	7.27	2.51
WSR_100_	16.26	16.26	16.13	15.93	14.58	15.56	15.49	15.62	15.59	15.54	15.49	13.88	12.89	12.16	N.S
PM_50_ + WSR_50_	13.84	13.80	13.73	13.67	13.59	13.56	13.35	13.14	12.88	12.47	11.86	11.45	11.15	10.99	N.S
UN_100_	6.52	6.49	6.46	6.44	6.40	6.38	6.31	6.25	6.16	5.79	5.46	5.48	5.35	5.30	N.S
UN_50_ + PM_50_	8.77	8.72	8.70	8.69	8.67	8.65	8.60	8.42	8.15	7.89	7.46	7.26	7.10	6.82	1.40
UN_50_ + WSR_50_	11.59	11.52	11.50	11.48	11.37	11.36	11.13	10.99	10.56	10.18	9.92	9.65	9.39	9.22	N.S
UN_50_ + PM_25_ + WSR_25_	10.18	10.15	10.13	10.08	10.01	9.99	9.86	9.68	9.43	9.16	8.90	8.54	8.30	8.17	N.S

Tukey’s HSD (*P* ≤ 0.05)	4.56	4.48	4.49	4.14	4.24	3.40	4.41	4.20	3.93	2.27	2.91	2.74	2.65	1.90	

*PM, poultry manure; WSR, wheat straw residues; UN, urea N. PM_*100*_, WSR_*100*_ and UN_*100*_ means application of these amendments alone with full dose at the rate of 200 mg N kg^-*1*^ soil. The subscripted values used (i.e., 100, 50, and 25) on each source (PM, WSR, and UN) refer to the percentage of total N applied from each source. The Tukey ‘s HSD (*P* ≤ 0.05) at the end of each row represent significance level among different incubation periods.*

The timings effect indicated that, SOC of different amendments continuously decreased over time (except for PM_50_ + WSR_50_). By the end of incubation at d 140, the relative reduction in SOC ranged between 19 and 34% over their initial SOC at day 0. The SOC unaccounted for may be associated with higher microbial activities that utilized SOC as an energy source or possible C loss from microbial decomposition or C-mineralization. Under field conditions, regular additions of organic materials in soils for longer periods had been reported to improve the level of organic C (Blair et al., 2006; Cavigelli and Dao, 2008; Abbasi and Tahir, 2012). However, under laboratory conditions for short-term periods without plant biomass, loss of SOC is expected because of C and N mineralization of added organic materials.

The SOC under different amendments varied between 8.14 and 15.1 g kg^-1^ compared to 6.05 and 6.06 g kg^-1^ in the control and UN_100_ treatments, respectively, representing a relative increase of 35 to 150% over the control and UN treatment (**Table 6**). The sequestration of SOC was highest in organic amendments [12.34 g kg^-1^ (as a group)] followed by integrated treatments (9.44 g kg^-1^), demonstrating a relative increase of 104 and 56%, respectively. The variability in SOC among different amendments supplemented either with PM or WSR may be attributed to the differences in the quality of added materials such as C content, C:N ratio, lignin, and polyphenol contents (**Table 2**). In addition, the variation in mineralization potential may have affected the accumulation of SOC as observed in WSR that had low N mineralization but high C accumulation.

## Conclusion

Poultry manure and WSR when applied alone or in combination with urea N, improved organic matter buildup and increased available and total N concentration of soil. The quantitative measurements of a substantial amount of potentially mineralized N from these organic sources demonstrated the significance of these materials as value-added nutrient inputs. However, slow rates of mineralization in the initial stage and low nutrient contents are the main limitations associated with the use of these amendments as major plant nutrient sources. This limitation can be overcome by the integrated use of these organic materials with minimum mineral N fertilizers. It is worth noting that N released from the urea N continuously declined after day 6, and after 140 days, 37% of N was unaccounted for. In contrast, application of urea with PM and/or WSR continuously released N into the mineral N pool without showing any declining trend indicating the persistence and retention of N in soil mineral pool for a longer period. These results suggest a judicious N application strategy through integration of both organic and inorganic sources for improving current N supplying capacity of soil and maintaining soil organic C pool in the soil.

## Author Contributions

AK did the Research work while MKA design the experiment and writing up the manuscript.

## Conflict of Interest Statement

The authors declare that the research was conducted in the absence of any commercial or financial relationships that could be construed as a potential conflict of interest. The reviewer AY and handling Editor declared their shared affiliation, and the handling Editor states that the process nevertheless met the standards of a fair and objective review.

## Footnotes

1 www.SPSS.com
